# Evolution of *Escherichia coli* strains under competent or compromised adaptive immunity

**DOI:** 10.1371/journal.ppat.1012442

**Published:** 2025-04-24

**Authors:** Camille Ameline, Elsa Seixas, Hugo C. Barreto, Nelson Frazão, Miguel V. Rodrigues, M. Rita Ventura, Marta Lourenço, Isabel Gordo

**Affiliations:** 1 GIMM - Gulbenkian Institute for Molecular Medicine, Evolutionary Biology, Lisboa, Portugal; 2 Université Paris Cité, CNRS, Inserm U1016, Institut Cochin, Paris, France; 3 Universidade Católica Portuguesa, Faculdade de Medicina, Centro de Investigação Interdisciplinar em Saúde, Lisboa, Portugal; 4 Instituto de Tecnologia Química e Biológica António Xavier, Universidade Nova de Lisboa, Oeiras, Portugal; St Jude Children's Research Hospital, UNITED STATES OF AMERICA

## Abstract

*Escherichia coli* is a commensal of the intestine of most mammals, but also an important human pathogen. Within a healthy human its population structure is highly dynamic, where typically a dominant *E. coli* strain is accompanied by several low abundance satellite strains. However, the factors underlying *E. coli* strain dynamics and evolution within hosts are still poorly understood. Here, we colonised germ-free immune-competent (wild-type) or immune-compromised (*Rag2KO*) mice, with two phylogenetically distinct strains of *E. coli*, to determine if strain co-existence and within-strain evolution are shaped by the adaptive immune system. Irrespectively of the immune status of the mice one strain reaches a 100-fold larger abundance than the other. However, the abundance of the dominant strain is significantly higher in *Rag2KO* mice. Strains co-exist for thousands of generations and accumulate beneficial mutations in genes coding for different resource preferences. A higher rate of mutation accumulation in immune-compromised *vs.* immune-competent mice is observed and adaptative mutations specific to immune-competent mice are identified. Importantly, the presence of the adaptive immune system selects for mutations that increase stress resistance and the dynamics of such evolutionary events associates with the onset of an antibody response.

## Author Summary

*Escherichia coli* is both a gut commensal but also an important human pathogen. A healthy human typically hosts more than one strain of *E. coli* in its intestine. We currently have limited knowledge about the factors governing the abundance and evolution of different *E. coli* strains within hosts. Here, we colonised immune-competent (wild-type) or immune-compromised (*Rag2KO*) mice devoid of a complex microbiota, with two distinct strains of *E. coli*. We find that both strains co-exist at different abundances for thousands of generations in the gut of both hosts, and that the evolutionary changes that occur in each strain are shaped by the mouse adaptive immune system. During the thousands of generations of co-existence, strains accumulate beneficial mutations that alter their resource preferences, and a higher rate of mutation accumulation in immune-compromised *vs.* immune-competent mice is observed. Importantly, specific mutations that increase stress resistance were selected only in the presence of the adaptive immune system and the dynamics of such evolutionary events associates with the onset of an antibody response.

## Introduction

*E. coli* is one of the first facultative anaerobes to colonize the mammalian gut. It can alter the physiology of this organ in newborns, making it a relevant commensal of the microbiota since birth [1]. Despite the positive influence of *E. coli* in early gut colonization, *E. coli* is also responsible for a high mortality in children (75 million episodes of diarrhea in children under 5 years of age, leading to tens of thousands of deaths) [2]. Furthermore, it is considered a risk factor for necrotizing enterocolitis [3], and its expansion in the intestine of adults has also been observed in inflammatory disease conditions, such as in patients with inflammatory bowel disease (IBD), and in the elderly. The expansion of *E. coli* was also shown to occur in clinical and animal studies, with blooms of *E. coli* frequently observed in both mice and humans [4].

In humans, a few longitudinal studies have shown that *E. coli* strain diversity is highly variable [5–7]. However, the causes underlying *E. coli* genetic variation and the virulence of certain strains are still unclear. What factors cause the observed diversity and persistence of *E. coli* strains within a host (be it a human or a mammal), and how these relate to *E. coli* pathogenesis is still a big mystery [8].

*E. coli* is known to undergo rapid evolution when colonizing the gut of laboratory mice [9–12]. We have previously shown that the gut ecology and evolution of an *E. coli* strain when colonizing mice depends on the composition of the other microbiota members [13,14]. Using a classical mouse colonization model, the streptomycin-treated mouse [15], we have also shown that the speed of evolution of this gut commensal can differ in immune-competent (wild-type) *vs.* immune-compromised mice (*Rag2KO*), and those differences reflect differences in microbiota composition in these distinct hosts [16].

Specific mouse-*E. coli* colonization models have shown that the adaptive immune system can be primed by *E. coli* and react to this bacterium when it colonizes the gut at high abundances (> 10^8^ cells/g) [17]. Furthermore, it was recently found that *E. coli* elicits a range of antigen-specific immunoglobulins (IgAs) targeting defined surface and non-surface membrane antigens; and that the intestinal antibody response to gut colonizing *E.coli* causes alterations in the expression of traits of this bacteria (carbon-source uptake, phage resistance, motility and membrane integrity) that are considered important for virulence and antibiotic resistance phenotypes [18].

Here, we study the eco-evolutionary dynamics of two *E. coli* strains, belonging to distinct phylogenetic groups, when they colonize the intestine of immune-competent (wild-type) or immune-compromised (*Rag2KO*) germ-free mice. We aim at testing the hypothesis that the adaptive immune system shapes the evolution of *E. coli* strains, independently of any other members of the gut microbiota. *Rag2KO* mice lack mature T and B lymphocytes and as such may represent an environment where the selective pressure over commensal bacteria may be reduced. In these mice we expect the abundance of *E. coli* to be higher than in wild-type mice and the pattern of evolution to be dominated by adaptations to the gut metabolic environment. In wild-type mice we expect the selective pressure driven by the adaptive immune system to be higher than in *Rag2KO*, due to the coating of bacteria by immunoglobulins that contribute to the elimination of bacteria [19]. If such pressure is sufficiently strong, then we would expect the pattern of *E. coli* evolution to be marked by specific adaptations dependent on the adaptive immune system.

## Results and discussion

### Co-existence of *E. coli* strains and higher abundance in immune-compromised mice

**Fig 1:**
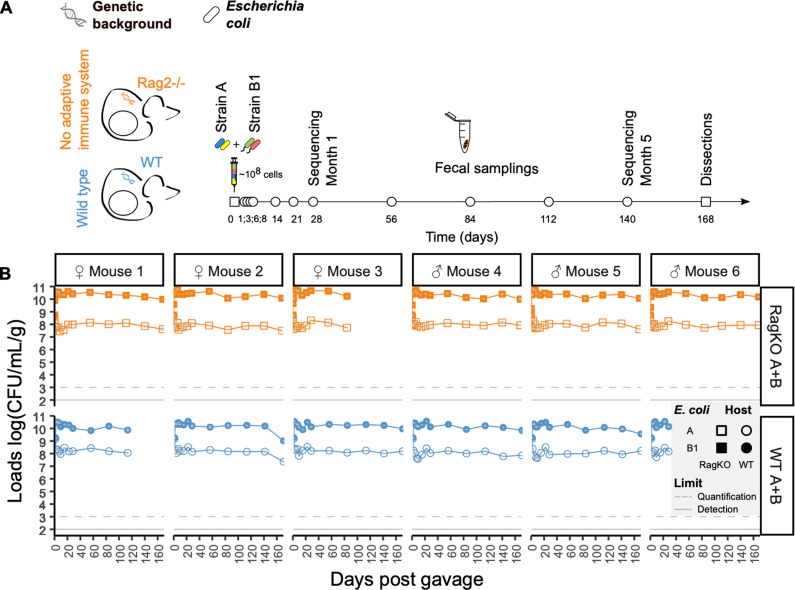
Co-existence of phylogenetic distinct strains of *E. coli* in immune-competent (WT) or immune-compromised (*Rag2KO*) germ-free mice. A) Design of experimental evolution in wild-type and Rag2KO mice. B) Irrespectively of the host genetic background both strains (A and B1) can coexist during thousands of generations (160 days correspond to 3040 generations of *E. coli* in the gut of WT mouse and 3520 generations in the gut of *Rag2KO* mice). Grey bars represent 2 s.e.m. Mice *Rag2KO* number 3 and wild-type mice 1 and 6 died of natural causes before day 168.

To study how the adaptive immune system shapes the evolution of commensal strains, we colonized wild-type and *Rag2KO* germ-free mice (n=6 for each mouse strain) with two strains of *E. coli*. One of the strains belongs to phylogenetic group A (lab-adapted strain A, hereinafter referred to as strain A) and the other belongs to phylogenetic group B1 (mouse commensal strain B1, hereinafter referred to as strain B1) (Fig 1A). Strain A is commonly used in studies of laboratory adaptive evolution (e.g. [20–22]) and in studies of adaptation during colonization of the mouse gut (e.g. [9,23]). It is known to undergo rapid adaptation during the first weeks of gut colonization of either germ-free mice [14,23] or of mice with a complex microbiota [10,16]. Strain B1 is a typical resident of the intestinal tract of laboratory mice, and its rate of evolution in mouse gut is still poorly known [24,25]. We labeled each strain with two neutral fluorescence markers (Fig 1A) that allow for a rapid assessment of intra-strain evolutionary change (see below) and followed their abundances in fecal samples, taken from both host genotypes, throughout six months of colonization (Fig 1B). In both mouse genetic backgrounds the two strains co-exist for several thousand generations (*E.coli* experiences ~19 generations per day in germ-free WT mice and 22 generations per day in *Rag2KO* mice [16]), but the mouse commensal strain B1 reaches 100-fold higher abundance than the lab-adapted strain A (Fig 1B and S1 Table). Dominance of the mouse commensal strain B1 over strain A is also observed along all sections of the intestine after 169 days of colonization in WT and 174 days of colonization in *Rag2KO* mice (S1 Fig and S2 Table). Remarkably, we also find that the abundance of strain B1 is significantly higher in *Rag2KO* mice than in wild-type mice (F=19.6, P=0.00002, General Linear Model with repeated measures Anova, S2A Fig), whereas the oppositive pattern occurs for the strain A (F=24.8, P= 0.0005, S2A Fig). It was previously demonstrated that germ-free wild-type mice can mount an IgA response against *E. coli*, when it reaches colonization loads above 10^8^ per g/feces [17]. Consistent with this notion we confirmed the presence of high levels of IgA in wild-type mice (S2B Fig and S3 Table). Since one of the functions of IgA is to coat bacteria and increase the chances that they are eliminated, induction of an IgA response towards the most abundant *E. coli* strain could contribute to its lower abundance in wild-type mice when compared with *Rag2KO*, which do not produce IgA. However, the abundance of the B1 strain is already higher in the first days of colonization, before a specific IgA response is induced, indicating that the strains differ in their engraftment capacities.

### Intra-strain polymorphism in immune-competent and immune-compromised mice

**Fig 2:**
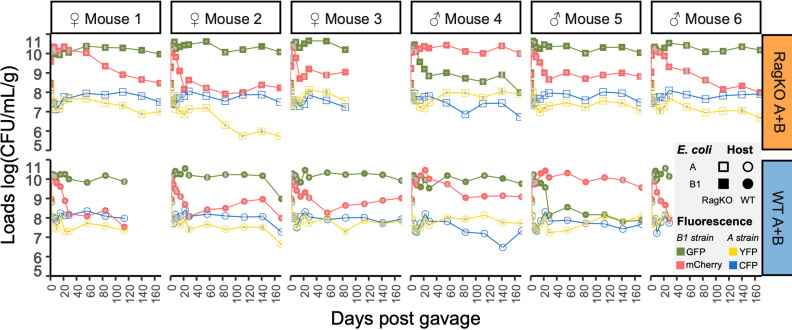
Maintenance of intra-strain polymorphism. The densities of each colonizing strain were estimated from faecal samples taken along time. Irrespectively of the host genetic background, variation at the neutral fluorescence locus (YFP or CFP for strain A; and mCherry or GFP for strain B1) is maintained in both strains, despite the substantial changes in the frequency of each marker. Vertical grey bars correspond to 2 s.e.m. Mice *Rag2KO* number 3 and wild-type mice 1 and 6 died of natural causes before day 168.

To understand the evolutionary dynamics of each *E. coli* strain we followed the frequencies of their neutral markers (Fig 2). If evolution is dominated by strong selective sweeps, caused by the emergence of mutations that rapidly fix, one would expect fixation of one of the fluorescence markers [26]. If a distinct mode of selection dominates the evolution (e.g. a form of selection which can maintain long-term polymorphism [24,27,28]) occurs, then both neutral markers should be maintained.

In both wild-type and *Rag2KO* mice we observed maintenance of the markers in both strains (Fig 2), suggesting that selective sweeps are not the dominant mode of selection. The maintenance of neutral polymorphism in the lab-adapted strain A in germ-free wild-type mice is quite distinct from what is seen in microbiota-rich (specific pathogen free, SPF) mice [29]. When SPF mice are co-colonized with the same strains of *E. coli*, the lab-adapted strain A loses of one of the fluorescent markers in less than 1500 generations of evolution in the gut, whereas maintenance of the markers occurs only in the mouse commensal strain B1[29]. This strongly suggests that the other microbiota members influence the dynamics of evolution of *E. coli* strains that coexist in the gut. While it was previously shown that the microbiota influences the speed of adaptation of a single gut colonizing *E. coli* strain [14], to the best of our knowledge this is the first time that a strain specific effect in the dynamics of *E. coli* evolution modulated by the microbiota is seen.

### Horizontal gene transfer in immune-competent and immune-compromised mice

**Fig 3:**
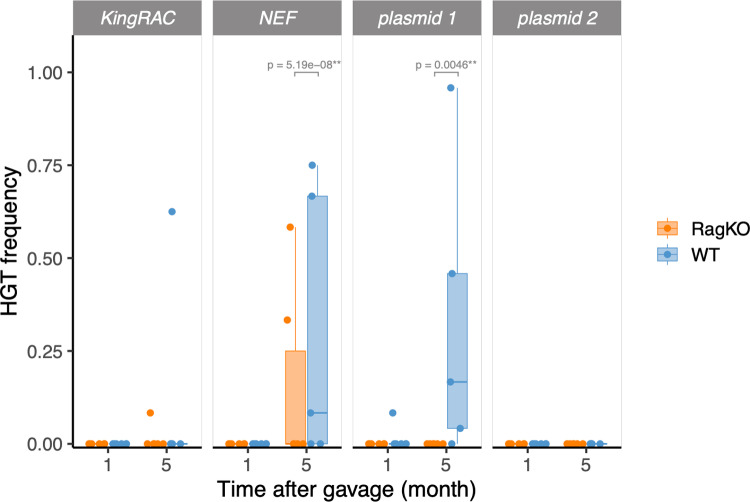
Horizontal transfer from mouse commensal strain B1 to lab-adapted strain A. The transfer of prophages Nef and KingRac and of the two plasmids carried by strain B1 was measured by PCR in clones of strain A (n~20) after one month or five months of evolution in the gut. Very few transfer events were detected in either wild-type or *Rag2KO* mice after the first month of colonization, but after five months transduction and conjugation events can be detected.

The coexisting strains are quite distinct in their genomes and in mobile genetic element content, with strain B1 carrying two conjugative plasmids and the potential to transfer extensive amounts of DNA [25]. Furthermore, previous results from our lab showed that phage-driven horizontal gene transfer from the mouse commensal strain B1 to the lab-adapted strain A can rapidly occur in wild-type mice with a complex microbiota (SPF mice) [24,29] and also in ageing mice that have a less effective immune system [30]. To determine if the adaptive immune system influences genetic exchange between the strains in the absence of other components of the microbiota, we typed evolved clones of the lab-adapted strain A for the presence of elements previously shown to be transferable between the strains, using PCR (Fig 3). After one month of colonization, no significant difference in the number of typed HGT events was observed between wild-type and *Rag2KO* mice. In the long-term (month 5, day 140), we found a significantly higher level of transfer of the prophage Nef in wild-type *vs. Rag2KO* mice, (Binomial GLM, p = 0.0046) and also a significant higher level of transfer of plasmid 1 in wild-type mice (Binomial GLM, p < 0.001). In a previous study [25] we found that the acquisition of the Nef prophage by strain A leads to an increased fitness relative to the ancestor (which does not carry Nef) during in vitro growth in minimal media with mannose or with gluconate, two carbon sources that are present in the gut [31]. In future studies it would be interesting to determine if the presence of a complex microbiota affects the rate of phage and plasmid transfer in wild-type and *Rag2KO* mice.

### Higher rate of evolution in immune-compromised mice in the initial period of adaptation

**Fig 4:**
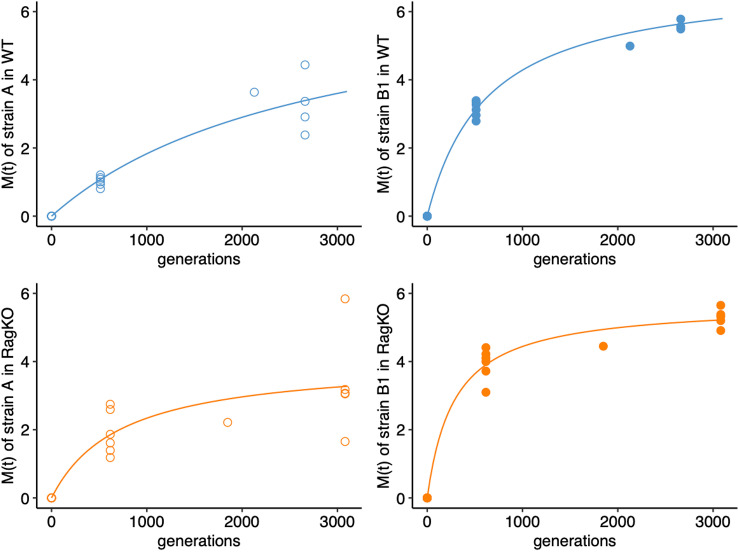
Mutation accumulation in wild-type and in mice.*Rag2KO* We quantified mutation accumulation along time (M(t)) as the sum of the mutation frequencies at each time point sequenced for each strain in each mouse (points in each panel). The fit of a hyperbolic function (h(t)=a*t/(*b*+t)) to the mean number of mutations accumulated along time for each strain in each mouse genetic background (wild-type (WT) mice in blue and *Rag2KO* mice in orange) is shown as a line. For the lab-adapted strain A in WT mice: *a*=6.97 (SE=2.1, P=5x10^-3^), *b*=2803 (SE=1498, P=8x10^-2^), R=0.96. For the strain B1 in WT mice: *a*=6.97 (SE=0.21, P=2x10^-15^), *b*=626 (SE=57, P=1.4x10^-8^), R=0.996. For strain A in *Rag2KO* mice: *a*=4.04 (SE=0.83, P=2x10^-4^), *b*=728 (SE=463, P=1.4x10^-1^), R=0.86. For strain B1 in *Rag2KO* mice: *a*=5.7 (SE=0.22, P=1.3x10^-14^), *b*=283 (SE=52, P=6.3x10^-5^), R=0.99.

To determine if the adaptive immune system influences the rate of evolution *via* mutation accumulation in each strain we performed pool-sequencing of clones that evolved in the mouse gut. For each mouse we estimated the rate of mutation accumulation per generation (M(t), by summing the frequencies of the detected mutations at a given sampling day and dividing it by the corresponding number elapsed generations (S4 Table). For strain A we find a higher rate of mutation accumulation in immune-compromised mice (T-test P=0.034) in the first month of colonization, while for strain B1 no significant difference was found (T-test P=0.52) in the first month of colonization. For the lab-adapted strain A, M(t) was 0.002 per generation in wild-type mice and 0.003 per generation in *Rag2KO* mice (Fig 4 and S5 Table). For the mouse commensal strain B1 M(t) after one month of colonization was 0.006 in wild-type mice and 0.0064 in *Rag2KO* mice (Fig 4 and S6 Table). Independently of the host genetic background, strain B1 evolved faster than strain A (Paired T-test P=0.000013 for wild-type mice, and P=0.0007 for *Rag2KO* mice). In a previous study we followed the evolution of strain A in wild-type and *Rag2KO* mice carrying a complex microbiota (SPF mice), but no other *E. coli* strain. After one month of colonization, we found that strain A evolved at a slower pace in *Rag2KO*, due to differences in microbiota composition between immune-competent and immune compromised mice [16]. In contrast, here we find a higher rate of evolution of strain A in immune compromised mice devoid of a complex microbiota. Together these results suggest that both the adaptive immune system and the composition of the microbiota influence the rate of evolution of a focal bacterial strain.

The Anna Karenina Principle predicts that the microbiomes of unhealthy individuals are much more distinct between one another than those of healthy ones [32,33]. Since *Rag2KO* mice are immune compromised we enquired if an Anna Karenina effect could be detected in the process of evolutionary adaptation of each strain. Interestingly, we find that the rates of mutation accumulation are significantly more variable in immune-compromised mice for strain A (F-test P=0.013), providing support for the Anna Karenina Principle in this less abundant strain. However, for the more abundant mouse commensal strain B1 no significant difference was detected (F-test P=0.32).

To understand if the differences in rates of mutation accumulation would be sustained in the long term, we pool-sequenced clones after more than 100 days of evolution. The mean number of mutations accumulated increased in both strains (Fig 4 and S5 and S6 Tables) but significant differences in their rates of evolution across host genetic backgrounds are no longer observed at these later time points. The dynamics of mutation accumulation is well fitted by a hyperbolic function, especially for the more abundant B1 strain, which implies that the rate of evolution declines with time in both wild-type and *Rag2KO* mice that do not have a complex microbiota (Fig 4). Just as observed in short term evolution, in the long term the dominant strain B1 accumulates more mutations than strain A in both hosts (Paired T-test P=0.0033 for wild-type mice, and P=0.013 for *Rag2KO* mice).

### Evolutionary parallelism within each strain across hosts

**Fig 5:**
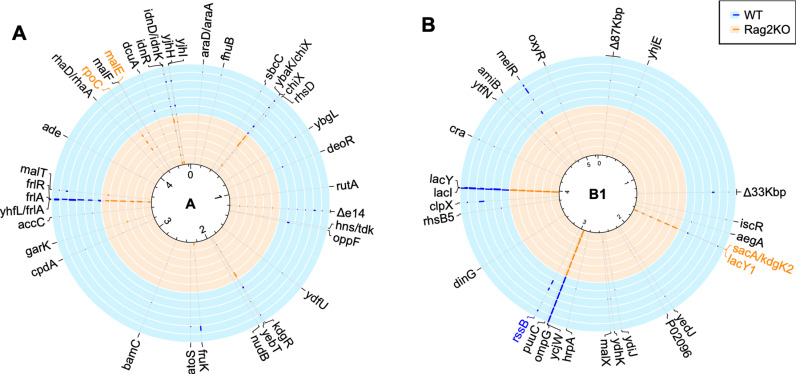
Targets of adaptation in wild-type and *Rag2KO* mice. A) Circular plots indicating the genes or intergenic regions where parallelism across mice was observed for the laboratory strain A. B) Circular plots indicating the genes or intergenic regions where parallelism across mice was observed for the mouse commensal strain B1. Within each circle (light blue for wild-type mice and light orange for *Rag2KO* mice) the maximum frequency of the mutations observed at any sampling point is indicated with a bar (ranging from 0 to 1). Highlighted in orange are the genes or intergenic regions where a significantly higher frequency of mutations occurred in *Rag2KO* mice (in strain A, T-test P=0.039 for *rpoC* and P=0.0499 for *malE*; in strain B1, T-test P=0.000014 for *lacY1* and P=0.05 for *sacA/kdgK2*). Highlighted in blue is the gene where a significantly higher frequency of mutations occurred in wild-type mice (*rssB*, T-test P=0.05). Intergenic mutations near the promoter region were considered as affecting the gene under the promoter regulation. Circular plots were made with R package circlize [37].

To determine how the adaptive immune system shapes the adaptive landscape of each strain, we analyzed the level of mutational parallelism of the sequenced clones at the gene target level. Mutations at the same target, that evolve independently in more than one animal (parallel mutations), are a strong indication of adaptive evolution [34]. The probability of parallelism is known to depend on population size. In large populations the supply of rare strong effect beneficial mutations that can increase in frequency by selection is expected to be higher in large than in small populations. Even if competition between clones carrying distinct beneficial mutations occurs in such large populations, higher parallelism should still be seen [34]. Thus, in our experiments we would expect that the level of parallel evolution should be higher in the more abundant strain (mouse commensal strain B1) compared with the less abundant strain (lab-adapted strain A). Furthermore, theoretical models of adaptation towards a single fitness peak landscape predict that the probability of parallel evolution should increase with decreasing distance of the population to the fitness peak [35,36]. Thus, if the mouse commensal strain B1 is closer to the fitness peak than the lab-adapted strain A, then the level of parallel evolution should be higher in strain B1 than strain A. In strain A we detected mutations in 225 genetic targets (S5 Table) and in 36 of these, parallelism occurred (Fig 5A). In strain B1 we detected mutations in 282 genetic targets (S6 Table) and in 28 of these, parallelism occurred (Fig 5B). Thus, in contrast to the theoretical expectations detailed above, the probability of parallel evolution at the gene level was higher for the lab-adapted strain (16% for strain A vs. 9.9% for strain B1). Large differences in genome size between the strains (4.6Mbp strain A *vs*. 5.2 Mbp strain B1) could potentially contribute to the observed differences in the frequency of genetic parallelism. Another possible contribution to our results is the fact that the mouse commensal strain B1 was isolated from a mouse with a complex microbiota, thus adapted to an environment in which other bacterial species are present.

### Intragenic clonal interference in immune-competent and immune-compromised mice

Within each mouse there was evidence for multiple alleles segregating at the same locus. This indicates that a high level of intragenic clonal interference marked the intra-host adaptation of *E. coli*. For the less abundant strain (A), this occurred in 7 genes (S3 Fig*): frlR*, a fructoselysine-6-phosphate specific repressor [38], which was previously shown to acquire adaptive mutations in the gut [24], and the transporter of this carbon source, *frlA* [39]; *chiX*, a small RNA whose expression was downregulated in the presence of chitosugars [40]; *fruK*, a kinase essential for utilization of fructose as a carbon source [41]; *kdgR*, a 2-Keto-3-deoxygluconate repressor (previously shown to acquire adaptive mutations in the gut [24]); *garK*, a kinase induced when *E.coli* grows on glycolate, glucarate, glycerate, and galactarate [42]; and *oppF*, a murein tripeptide ABC transporter [43].

For strain B1, we found evidence of intragenic clonal interference in 10 genes (S3 Fig). This was particularly remarkable for the lactose operon (*lacI*, *lacY*, *lacY1*), for the outer membrane protein G precursor *ompG* and for the HTH-type transcriptional repressor *purR*, which showed very high level of parallelism across immune-competent and immune-compromised mice. Interestingly, the Lac operon and genes *ompG*, and *purR* although described as having distinct roles in bacterial physiology, can potentially be functionally connected due to their involvement in metabolic regulation, nutrient uptake, and environmental response in *E. coli*. The Lac Operon, famously known for the uptake and metabolism of lactose, was recently shown to also be used by *E. coli* in the gut to consume raffinose [9]. *OmpG* encodes an outer membrane protein in *E. coli*. When expressed, *ompG* functions as a nonspecific outer membrane porin and in addition to the efficient transport of monosaccharides, *ompG f*acilitates the diffusion of dissacharides (sucrose) and trisaccharides (raffinose) [44]. On the other hand, *purR* is a transcriptional repressor belonging to the GalR/LacI family and is involved in the regulation of purine biosynthesis pathways [45]. Metabolic interactions between lac operon, *ompG*, and *purR* could be considered since Lac metabolism influences energy levels and metabolite availability, which can potentially impact pathways regulated by *purR* or transport mediated by *ompG*. We hypothesise of a possible functional link, since *ompG* facilitates the diffusion of raffinose, and the Lac Operon is in turn used by *E. coli* in the gut to consume raffinose. Moreover, *ompG*, being a porin, contributes to nutrient uptake in low-nutrient conditions. If lactose or purines are among the available nutrients, *ompG*-mediated transport could indirectly influence the activity of Lac or *purR* pathways. At the same time, both LacI and PurR are transcriptional repressors in *E. coli* and control various important metabolic pathways. For example, changes in nucleotide levels (PurR’s domain) could impact Lac operon activity if the cell’s preference for carbon sources shifts.

The parallel adaptations in the fructoselysine operon observed in strain A and in the lactose operon observed in strain B1, is compatible with evolution towards specialization to distinct resources under co-colonization of the gut. Interestingly, strain B1 lacks the fructoselysine operon, so it cannot evolve to specialize on this resource, and strain A lacks the lactose operon. Growth assays on minimal media supplemented with raffinose, which is present in the gut of germ-free mice and can be utilized by *E.coli* [9], corroborated that evolved clones of strain B1 have an increased fitness in this carbon source compared with its ancestor (S6B Fig). Furthermore, evolved clones of strain A also showed an increased fitness when grown in minimal media supplemented with fructoselysine (S6A Fig). Importantly, evidence for intense selection in the *rssB* gene was detected in a host genotype specific manner. The RssB protein is a major regulator of the global stress response factor σ^s^ (encoded by *rpoS*). It delivers σ^s^ to the protease ClpXP for degradation [46]. Multiple mutations in this gene, both within and across hosts (S3 Fig), were specifically detected in the mice with an adaptive immune system. Interestingly, mutations in *rpoS* and *clpX* were also detected specifically in immune-competent mice (S6 Table), suggesting that the regulation of the stress response was under selection in wild-type but not in *Rag2KO* mice.

### Increased stress resistance evolves in the presence of the adaptive immune system

**Fig 6:**
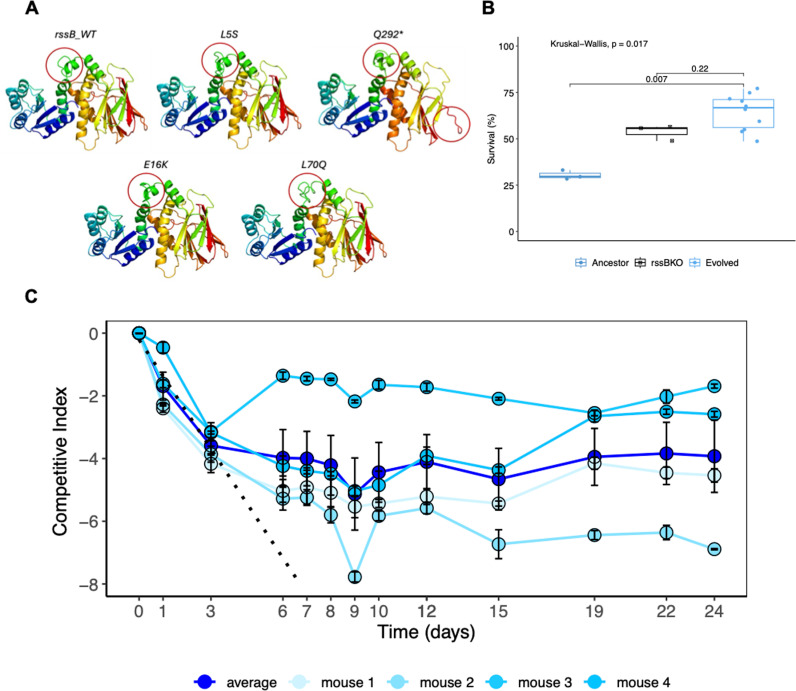
A) Predicted structures of the mutants of *rssB* detected in immune-competent mice compared with the structure of the ancestral *rssB* (differences highlighted with a red circle). B) Survival under oxidative stress of clones evolved in immune-competent mice. Ten evolved clones of strain B1, sampled from mouse WT5 at day 150, were tested for increased survival relative to their ancestor. The survival of a clone with a KO at *rssB*, which is known to have increased survival under stress, is also shown. C) Competitive Index (measured as the log of the ratio of *rssB*KO CFUs/g to wild-type *rssB* CFUs/g) of the *rssB*KO of strain B1 during 24 days of colonization of immune-competent mice (n=4). Dark blue circles represent the mean CI across mice, while light blue circles shows the CI for each individual mouse. Error bars represent the standard error. The KO of *rssB* is a deleterious mutation during the initial days of competition, and *rssB*KO clones should go extinct after 8 days of gut colonization (see the dashed line which represents the predicted dynamics of the *rssB*KO from the linear regression of the mean CI values during the first three days of competition). However, *rssB*KO clones resist extinction and are maintained in the gut, showing that they are positively selected for after the 1^st^ week of colonization, when the levels of IgA increase (S9 Table).

The signature of adaptive evolution in stress response genes was specific to the wild-type mice and to the dominant strain B1. The nature of some of the mutations in *rssB* is suggestive of a gain of resistance to stress conditions. In mouse WT5, three different SNPs were detected (L5S, E16K and Q292*), one being a stop codon; in mouse WT1, three other SNPs in *rssB* (S86P, L70Q and G40R) and one SNP in *clpX* (N94K) occurred; in mouse WT2 a *rssB* (G40R) mutation and a *rpoS* (V177F) mutation increased in frequency; in mouse WT4 a *rssB* (G31R) mutation reached 42% frequency and in mouse WT3 an amino-acid replacement in *clpX* (H95Y) reached 74% frequency. Bioinformatic analysis of the effect of changes on protein structure and function at the specific residues revealed the following predictions: all mutations detected in mouse WT5 are predicted to cause a conformation change in the linker region of the protein, which has been shown to play a critical role in the turnover of sigma factor S (rpoS) [47] (Fig 6A), and a similar conformational change occurs for the *rssB* variants evolved in mouse WT1(Fig 6A). Such structural change is likely to result in a change in phenotype (see S4 Fig and S1 Text). We therefore performed phenotypic tests of the evolved clones *in vitro*, under oxidative stress, and compared them with the phenotype of a *rssB* KO. We find that clones evolved in the gut of wild-type mice show higher survival than their ancestor and a survival under stress similar to that of a *rssB* KO (Fig 6B and S7 Table).

The mutations causing increased resistance to stress were only observed in strain B1, which due to its higher loads could stimulate the production of immunoglobulins in the mice with adaptive immune system [17]. If adaptive mutations in *rssB* are driven by the adaptive immune system when *E. coli* is in a high abundance, then we expect that in mice colonized solely with strain A mutations in *rssB* should also be detected. This is indeed what is observed in two previous studies, where wild-type germ-free mice were mono-colonized with strain A, which reached abundances well above 10^8^/g [9,14]. In Barroso-Batista et al [14] *rssB* mutations were detected at day 23 or 40 post-colonization in 6 out of 10 wild-type mono-colonized mice. Most of the mutations observed were IS insertions into *rssB*, consistent with a loss of function for this gene with the consequent increase in stress resistance. In the experiments of Vasquez et al [9], a barcoded strain A was used to mono-colonize germ-free mice and pool sequencing of evolving clones was performed for approximately 40 days. Using their sequencing data, we can detect *rssB* mutants in 10 out of 15 mice and follow their trajectories throughout the time period studied. Remarkably, *rssB* mutants only reached detectable frequencies after the first week of evolution (S5 Fig), consistent with the latency time to produce immunoglobulins by the adaptive immune system of the mice. To directly measure the fitness effect of *rssB*KO during gut colonization we performed a competitive fitness assay in wild-type mice. We found that the KO of *rssB* of strain B1 is deleterious during the initial days of competition but not after the first week of colonization (Fig 6C and S8 Table), consistent with positive selection on *rssB*KO clones after the onset of an adaptive immune response. Indeed, when we measure the concentration of IgA along time in the four mice where the competitive fitness assays were done, we find a significant increase of IgA along the days of competition (Spearman correlation r_S_= 0.84, P=0.00064) S7 Fig). In future experiments it would be interested to compare the eco-evolutionary dynamics of strains between wild-type and IgA KO mice to improve our understanding on how specific immune responses drive adaptation in the gut.

## Conclusion

Multiple strains of *E. coli* typically colonize the intestines of their hosts at different abundances. To understand how the adaptive immune system influences the evolution of co-existing strains of *E. coli* we performed *in vivo* experimental evolution in the gut of wild-type and *Rag2KO* mice. We found that, independent of host genotype, the strains evolved by acquiring mutations in genes involved in the consumption of different resources and this pattern of metabolic evolution was remarkable similar across all the mice. Importantly, we also found that mutations in the global stress response pathway of *E. coli* increased in frequency only in mice with an adaptive immune system and specifically in the dominant strain of *E. coli*. These results suggest that the adaptive immune system exerts a strong selective pressure over abundant bacteria strains.

## Materials and methods

### Ethics Statement

This research project was ethically reviewed and approved by the Ethics Committee of the Instituto Gulbenkian de Ciência (license reference: A009.2018), and by the Portuguese National Entity that regulates the use of laboratory animals (DGAV - Direção Geral de Alimentação e Veterinária (license reference: 008958). All experiments conducted on animals followed the Portuguese (Decreto-Lei nº 113/2013) and European (Directive 2010/63/EU) legislations, concerning housing, husbandry and animal welfare.

### *Escherichia coli* strains

Each of the *E. coli* strains used express fluorescent proteins and carry antibiotic resistance markers. The laboratory *E. coli* strain A is a derivative of K12 and expresses either a Yellow or a Cyan Fluorescent Protein, YFP or CFP, respectively. Each strain is also streptomycin resistant, and carries ampicillin (YFP) or chloramphenicol (CFP) resistance markers. The strains are MG1655 DM08-YFP gatZ::IS1 [galK::amp (pZ12)::PLlacO-1-YFP, strR (rpsl150), ΔlacIZYA::scar] and MG1655 DM08-CFP gatZ::IS1 [galK::cm (pZ12)::PLlacO-1-CFP, strR (rpsl150), ΔlacIZYA::scar] [10]. This strain does not contain plasmids, nor functional prophages. *E. coli* strain B1, a mouse commensal [25], expresses either a Red (mCherry) or a Green Fluorescent Protein (sfGFP), a chloramphenicol resistance marker and is lactose positive. This strain contains two conjugative plasmids that can be transferred in the gut and two temperate prophages that can be active in the gut [24]. The ΔrssB mutant of *E. coli* B1, was generated by lambda Red-mediated homologous recombination, using pKD46 plasmid (arabinose-induced). The *rssB* gene was substituted by a kanamycin cassette. The gene knockout was confirmed by directed PCR.

*E. coli* clones were grown at 37°C under aeration in liquid media Lysogeny Broth (LB) from SIGMA and LB agar plates. Media were supplemented with antibiotics streptomycin (100 µg/mL), ampicillin (100 µg/mL) or chloramphenicol (30 µg/mL) when specified. Serial plating of 1X PBS dilutions of feces in LB agar plates supplemented with the appropriate antibiotics were incubated overnight and YFP, CFP, mCherry and sfGFP-labeled bacterial numbers were assessed by counting the fluorescent colonies using a fluorescent stereoscope (Zeiss Stereo Lumar V12). After 168 days of colonization, mice were humanely sacrificed, and the different sections along the intestine were collected. The abundance of *E*. *coli* strains in the different sections collected, Duodenum, Jejunum, Ileum, Cecum, Colon and Rectum, was determined by plating serial dilutions of the sections in LB agar plates supplemented with the appropriate antibiotics. After overnight incubation, bacterial numbers were assessed by counting fluorescent colonies using a fluorescent stereoscope (Zeiss Stereo Lumar V12).

### Evolution experiment

We colonized germ-free mice (*Mus musculus*) strain C57BL/6 of two different genotypes: Wild-type (n=6, 3 females and 3 males) and Rag2KO (n=6, 3 females and 3 males) mice. Mice were supplied by the Rodent Facility at Instituto Gulbenkian de Ciência (IGC). We performed experimental evolution of the A and B1 *E. coli* strains (two color versions of each strain) when co-colonizing either Wild-type or Rag2KO germ-free mice of six to eight weeks of age.

We inoculated the four bacterial clones (two color versions of the two strains) from frozen stocks in 5 ml of Brain Heart Infusion broth supplemented with the appropriate antibiotics (streptomycin (100 µg/mL) for the A strain or chloramphenicol (30 µg/mL) for the B1 strain), and let them grow overnight with aeration at 37°C. The next day, we inoculated 20 uL of the first growth solution into 2 mL of BHI without antibiotics and adjusted bacterial density (OD600=2). We then prepared a bacterial suspension in 1X phosphate buffered saline (PBS) solution of ~10^8^ cells containing the four clones at equal amounts (1:1:1:1 mixture). We inoculated each mouse by gavage with 100 µL of the bacterial suspension after a 4-h starvation period of food and water, and housed them individually. We sampled their feces (one or two fecal pellets) during a period of six months and stored them in 15% glycerol at -80°C for later analysis.

### Competitive fitness of *rssB*KO in wild-type mice

The *E. coli* ancestral strain B1 (expressing mCherry) and a derived *rssB* KO clone (expressing GFP) were grown overnight in 5 ml of Brain Heart Infusion broth supplemented with chloramphenicol (30 µg/ml) from frozen stocks. The next day, bacterial density was adjusted (OD600=2), and a mixture of the two clones (1:1) was gavaged (~10^8^ colony forming units (CFUs)) into WT germ-free mice after a 4-h starvation period of food and water. Fecal pellets were collected over several days after gavage, and frozen at -80°C in 15% glycerol for assessment of the competitive fitness of the of the *rssB* KO clone.

For both the experimental evolution and the competitive assay, we measured bacterial load of the two fluorescently-labeled versions of both strains over time. Serial plating of 1X PBS dilutions of feces on LB agar plates supplemented with the appropriate antibiotics were incubated overnight and YFP, CFP, mCherry and sfGFP-labeled bacterial numbers were assessed by counting the fluorescent colonies using a fluorescent stereoscope (Zeiss Stereo Lumar V12).

Whole-genome sequencing and analysis pipeline.

DNA was extracted with Phenol-Chloroform from a pool of *E. coli* clones (n>1000) after plating fecal samples taken from each mouse on LB plates with antibiotic specific for each strain. DNA concentration and purity were quantified using Qubit and NanoDrop, respectively. The DNA library construction and sequencing were carried out by the IGC genomics facility using the Illumina Nextseq2000 platform. Raw sequencing reads were processed using *fastp* [48](version 0.23.2). Sequencing adapters were removed, and raw reads were trimmed bidirectionally by 4 bp window sizes, retaining an average base quality of 20. Reads shorter than 100 bp and those with more than 50% of bases having a Phred score below 20 were removed, followed by base correction of overlapping reads. References genomes for alignment of sequenced reads were K-12 substrain MG1655; Accession Number: NC_000913.2, for the alignment of reads from strain A; Accession Number: SAMN15163749 and for the alignment of reads from strain B1. Annotation of IS elements in the strain B1 genome was performed using ISEScan (version 1.7.2.3) [49], and only complete IS were considered for the annotation of the strain B1 reference genome. The annotated genomes for strain A and strain B1 are available on the GitHub platform at https://github.com/hugocbarreto/Lateral-gene-transfer-causes-genomic-repair-when-strains-coexist-in-the-gut. BBsplit (version 38.90) (https://www.osti.gov/servlets/purl/1241166) was used to remove reads with sequences highly divergent from the reference genome. Variant calling in the evolved populations was performed using the 0.37.1 version of the BRESEQ pipeline [50] with the polymorphism option on and default settings, except for: a) polymorphism minimum variant coverage of 5 reads; b) base quality cutoff of 30; c) minimum mapping quality of 20. To decrease the probability of detecting false positives in the evolved populations, sequencing reads from the ancestral clones were used as controls during variant calling. Variants predicted in the ancestral clones using the BRESEQ pipeline in polymorphism mode (using the parameters described above) were considered as false positives and excluded from the evolved population analysis. To further reduce the probability of detecting false positives in the evolved populations, variants in the ancestral clones that reached a frequency above 0.015 and were present in more than 3 reads were considered as false positives and removed from the evolved population analysis. To determine the frequency of prophage e14 excision in strain A, we first calculated the ratios between the e14 median coverage and the coverage in the left and right flank. The final frequency was obtained as the average of both ratios. Given the genomic proximity between the two mutations in *lacI* (+CTGG and Δ4 bp) detected in strain B1, the frequency of each mutation is inaccurately determined by BRESEQ. In the mice where both *lacI* mutations were present, the frequency of each allele was calculated as following: (1-Frequency of allele with higher number of reads)*(Frequency of allele with lower number of reads). All the remaining putative variants were verified manually in IGV [51], and the genome coverage for all populations was inspected for regions of missing coverage distinct from the ancestral clones. Manual inspection of genome coverage led to the identification of three large deletions in the strain B1 populations, and their frequency was calculated using a similar strategy as for the calculation of the excision of prophage e14. The custom R scripts used for this analysis are available at https://github.com/hugocbarreto/Lateral-gene-transfer-causes-genomic-repair-when-strains-coexist-in-the-gut).

### Horizontal Gene Transfer from strain B1 to strain A

The B1 *E. coli* strain contains two plasmids, characterized by the *repA* and *repB* genes, and two prophages, NEF and KingRAC, characterized by the *xerC* and *asd* genes, respectively [25]. The lab-adapted strain does not contain these elements.

PCR detection of ~69Kb (*repA*) and ~109Kb (*repB*) plasmids. Specific primers for the amplification of *repA* and *repB* genes, were used to determine the frequency of the 68935 bp (~69 Kb) and 108557 bp (~109 Kb) plasmids, respectively, in the evolving *E. coli* clones of strain A after month one and five post-colonization.

The primers used for *repA* gene were:

repA-Forward: 5’-CAGTCCCCTAAAGAATCGCCCC-3’ and repA-Reverse: 5’-TGACCAGGAGCGGCACAATCGC-3’.

For *repB* the primer sequences were:

repB-Forward: 5’-GTGGATAAGTCGTCCGGTGAGC-3’ and repB-Reverse: 5’-GTTCAAACAGGCGGGGATCGGC3’.

PCR amplification of plasmid-specific genes was performed in randomly isolated clones from the evolved *E. coli* populations. PCR reactions were performed in a total volume of 25 μL, containing 1 μL of each clone growth in liquid LB media, 1X Taq polymerase buffer, 200 μM dNTPs, 0.2 μM of each primer and 1.25 U Taq polymerase. PCR reaction conditions: 95ºC for 3 min, followed by 35 cycles of 95ºC for 30 s, 65ºC for 30 s and 72ºC for 30 s, finalizing with 5 min at 72ºC. DNA was visualized on a 2% agarose gel stained with GelRed and run at 160 V for 60 min.

PCR detection of the Nef and KingRac prophages. PCR amplification of phage-specific genes was performed as previously [25] to determine the frequency of lysogens in *E. coli* strain A.

Quantification of IgA by ELISA.

Total fecal IgA was measured by sandwich ELISA. For detection of total IgA, ELISA plates (Nunc) were coated with 5 µg/ml of goat anti-mouse IgA (Southern Biotech) capture antibody and incubated overnight at 4ºC. Plates were washed and blocked with 1% BSA in PBS for 1h at room temperature. Diluted samples and standard were added, and plates incubated for 2h at room temperature. Captured IgA was detected by horseradish peroxidase (HRP)-conjugated goat anti-mouse IgA antibody (Southern Biotech). ELISA plates were developed by TMB microwell peroxidase substrate (R&D Systems) and quenched by 1M H_2_SO_4_. Colorimetric reaction was measure at OD = 450 nm by a Microplate Reader.

Resistance to oxidative stress *in vitro.*

The test was performed *in vitro* with evolved clones and the *rssB* KO, under oxidative stress. The frozen stock of WT5 at day 150 after colonization was plated and the evolved clones were isolated. Assays were conducted on exponentially growing cells (OD_600_ between 0.15 and 0.30) cultivated in minimal media (M9 + 0.4% glucose). For oxidative stress, cells were exposed to 17.6 mM H_2_O_2_ (Sigma) added to the medium during 30 minutes, before inactivation by catalase (Roche, 2 µg ml^-1^). The percentage of survivors after exposure to stress conditions was measured by plating the bacteria directly onto LB plates and counting colonies after overnight incubation at 37ºC.

Protein Structure Analysis.

The predicted structure of the original rssB protein from *E. coli* B1 strain together with each one of the variant proteins was generated using phyre2 (Protein Homology/analogY Recognition Engine V 2.0) [52], a web-based tool for predicting and analysing protein structure and function (http://www.sbg.bio.ic.ac.uk/phyre2/html/page.cgi?id=index) [52]. Comparison between the variants and the original protein was performed in Dali protein structure comparison server (http://ekhidna2.biocenter.helsinki.fi/dali/) [53]. Structures used for Figs 6 and S4, were generated by phyre2.

Growth assays *in vitro*.

A pre-culture was prepared by inoculating single clones into 150 µl of minimal medium (MM) with 0,2% glycerol in a 96-well round bottom plate followed by incubation for 25 h at 37ºC with 600 rpm in a benchtop shaker (Thermo-Shaker PHMP-4, Grant). The OD600 of the single clone cultures was measured and adjusted to 0,05. To generate single clone growth curves the wells of a BioscreenC Honeycomb plate (Thermo Scientific) were filled with 300 µl of minimal medium with either 1 mM fructoselysine or 1% Raffinose culture medium and inoculated with the single clone cultures at an OD600 of 0,05. The OD600 was measured with the BioscreenC, (Oy Growth Curves Ab Ltd.) for 24 h at 37ºC with reading intervals every 10 minutes under continuous shaking with medium amplitude.

Chemical synthesis of Fructoselysine hydrochloride.

^1^H NMR spectra were obtained at 500 MHz in D_2_O, ^13^C NMR spectra were obtained at 126 MHz in D_2_O. Assignments are supported by 2D correlation NMR studies (COSY, HSQC, HMBC). Flash column chromatography: silica gel Merck 60, 0.040–0.063 mm (230–400 mesh). Analytical TLC: Aluminium-backed silica gel Merck 60 F254. Reagents and solvents were purified according to the literature [54,55]. Synthesis of Fructoselysine was adapted and optimised from previously published work by Delpierre *et al* [56]. A mixture of D-glucose (2.03mmol, 500 mg) and (*tert*-butoxycarbonyl)-L-lysine (2.03 mmol, 366 mg) in methanol (5 mL) was stirred for 5 h under reflux and inert atmosphere. The mixture was concentrated under vacuum and purified by flash chromatography (80:20 MeCN/H_2_O) and the isolated compound was dissolved in a 3M a HCl aqueous solution to remove the *tert*-butoxycarbonyl protecting group. The solution was concentrated by evaporation and filtered through activated carbon. The resulting aqueous solution was evaporated to dryness to afford fructoselysine hydrochloride (700 mg, 90% yield) as a yellowish-white solid. Using 2D correlation NMR studies, it was confirmed that fructoselysine was present as furanose form in aqueous solution.

^1^H NMR (500 MHz, D_2_O) δ 4.00 – 3.62 (m, 6H, fructose H-3, H-4,H-5, 2xH-6, lysine H-β), 3.26 (s, 2H, fructose H-1), 3.11 (dd, *J* = 9.5, 6.4 Hz, lysine H-ζ), 1.94 – 1.79 (m, 2H, lysine H-γ), 1.79 – 1.64 (m, 2H, lysine H-ε), 1.51 – 1.32 (m, 2H, lysine H-δ).

^13^C NMR (126 MHz, D_2_O) δ 174.6 (lysine C-α), 95.5 (fructose C-2), 69.6, 69.3, 69.0 (fructose C-3, C-4, C-5), 64.0 (fructose C-6), 54.6 (lysine C-β), 52.9 (fructose C-1), 48.1 (lysine C-ζ), 29.9 (lysine C-γ), 24.9 (lysine C-ε), 21.6 (lysine H-δ).

Statistical Analysis.

A General linear model with repeated measures Anova was used to analyze the temporal dynamics of the *E. coli* abundances, while colonizing the mouse gut. A Binomial Test was used to compare strain polymorphism proportions. A paired T-test was used to compare rates of mutation accumulation of each strain. A P-value < 0.05 was considered for statistical significance.

## Supporting information

S1 Fig Abundance of *E. coli* strains after six months of colonization in different sections of the gut of immune-competent (A) and immune-compromised (B) mice. The densities (colony forming units per g of sample) of each strain (empty symbol for lab-adapted strain A, filled symbol for mouse commensal strain B1) were measured after dissecting the intestine of mice at day 168 after colonization. At this time point four wild-type and five Rag2KO mice were still alive. Duo, Jej, Ile Cec, Col Rec stands for Duodenum, Jejunum, Ileum, Cecum, Colon and Rectum.
(TIF)

S2 Fig Abundance of dominant *E. coli* strain is higher in immune-compromised germ-free mice. **A)** After the first day of colonization, the loads of the strain B1 are significantly lower in WT (blue) than in Rag2KO (orange) (F=19.6 P=0.00002) and the loads of strain A are significantly higher in WT than in Rag2KO (DF=1 F=24.8 P= 0.0005, General linear model with repeated measures Anova). This data is similar to that in Fig 1. **B)** Concentration of IgA in mouse faecal samples taken at day 27 (circles), 112 (square) and 140 (triangles) after colonization with *E. coli*, measured via ELISA. As expected, IgA is produced in WT mice.
(TIF)

S3 Fig Targets for which evidence of intragenic clonal interference within each host is found. *rssB* is mutated in strain B1 specifically when colonizing immune-competent mice and shows a high number of multiple alleles segregating in two of mice.
(TIF)

S4 Fig Predicted structures of rssB protein of mutants of rssB detected in immune-competent mice, differences highlighted in red circle. Protein structure prediction was performed using phyre2.
(TIF)

S5 Fig Dynamics of rssB mutant frequencies in the experiment of Vasquez et al [9], where strain A is the sole colonizer of the gut of wild-type mice. (TIF)

S6 Fig Growth curves of ancestors and evolved clones (isolated from mouse WT3 at day 140). **(A)** Growth curves in media supplemented with fructoselysine of the ancestral and evolved clones of strain A. **(B)** Growth curves in media supplemented with raffinose of the ancestral and evolved clones of strain B1.
(TIF)

S7 Fig Concentration of IgA along time in the competition experiment between the rssBKO of strain B1 and the ancestor in immune-competent mice (n=4). Dark blue circles represent the mean IgA across mice, while light blue circles show the IgA concentration for each individual mice.
(TIF)

S1 Table Log10 of the abundances of mouse commensal strain B1 and lab-adapted strain A in WT and in *Rag2KO* mice (CFU/g of feces). (XLSX)

S2 Table Log10 of the abundances of mouse commensal strain B1 and lab-adapted strain A in WT along 6 sections of the gut of WT and *Rag2KO* mice at the end of the evolution experiment. (XLSX)

S3 Table Concentration of IgA measured in fecal samples. (XLSX)

S4 Table Mutation accumulation in mouse commensal strain B1 and in lab-adapted strain A in WT and in *Rag2KO* mice. (XLSX)

S5 Table Mutations detected during the adaptation of the lab-adapted strain A in WT and in *Rag2KO* mice. (XLSX)

S6 Table Mutations detected during the adaptation of the mouse commensal strain B1 in WT and in *Rag2KO* mice. (XLSX)

S7 Table Survival of ancestor and evolved clones under oxidative stress *in vitro*. (XLSX)

S8 Table Values of the competitive index of a *rssB* KO strain of the mouse commensal strain B1 in WT mice. (XLSX)

S9 Table Levels of IgA during the competitive fitness assays of the *rssB* KO strain of the mouse commensal strain B1 in WT mice. (XLSX)

S1 Text Supplementary Text. (DOCX)
