# Supplementary figures and images for: Evolution of Escherichia coli strains under competent or compromised adaptive immunity
Source: PLoS Pathog. 2025 Apr 24;21(4):e1012442. doi: 10.1371/journal.ppat.1012442 (PMC12021133; doi:10.1371/journal.ppat.1012442)

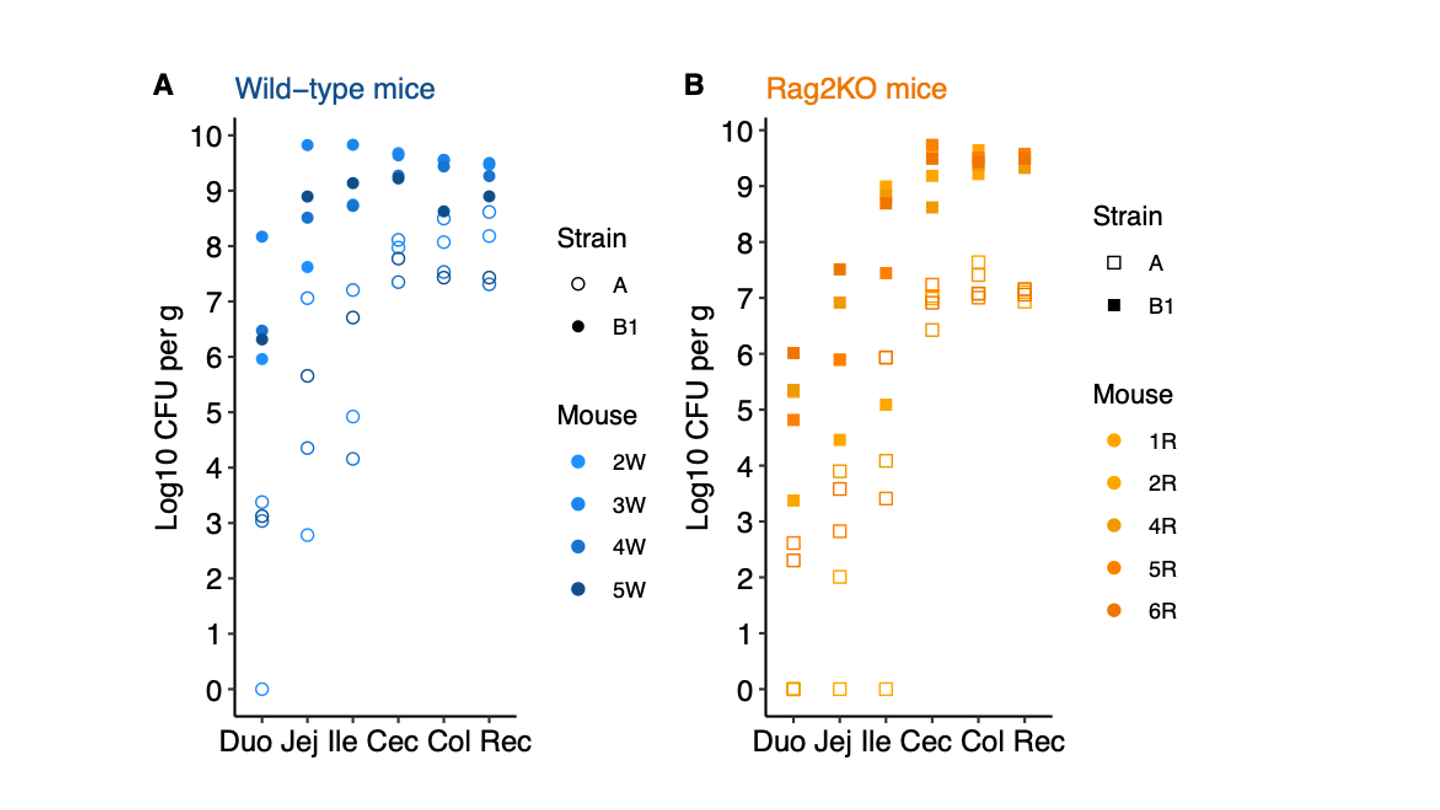

Supplement: S1 Fig — The densities (colony forming units per g of sample) of each strain (empty symbol for lab-adapted strain A, filled symbol for mouse commensal strain B1) were measured after dissecting the intestine of mice at day 168 after colonization. At this time point four wild-type and five Rag2KO mice were still alive. Duo, Jej, Ile Cec, Col Rec stands for Duodenum, Jejunum, Ileum, Cecum, Colon and Rectum. (TIF) [file ppat.1012442.s001.tif]

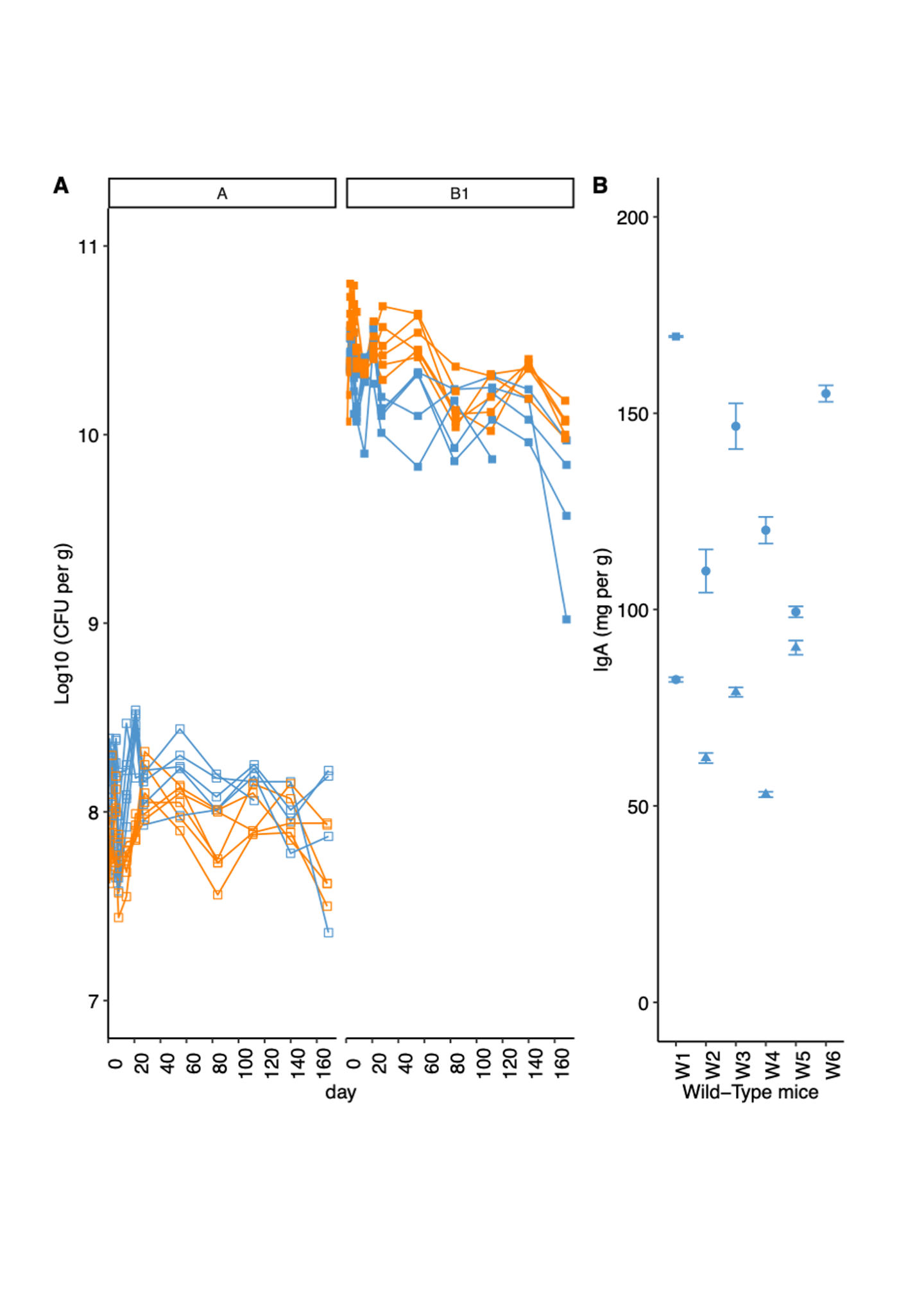

Supplement: S2 Fig — A) After the first day of colonization, the loads of the strain B1 are significantly lower in WT (blue) than in Rag2KO (orange) (F=19.6 P=0.00002) and the loads of strain A are significantly higher in WT than in Rag2KO (DF=1 F=24.8 P= 0.0005, General linear model with repeated measures Anova). This data is similar to that in Fig 1. B) Concentration of IgA in mouse faecal samples taken at day 27 (circles), 112 (square) and 140 (triangles) after colonization with E. coli, measured via ELISA. As expected, IgA is produced in WT mice. (TIF) [file ppat.1012442.s002.tif]

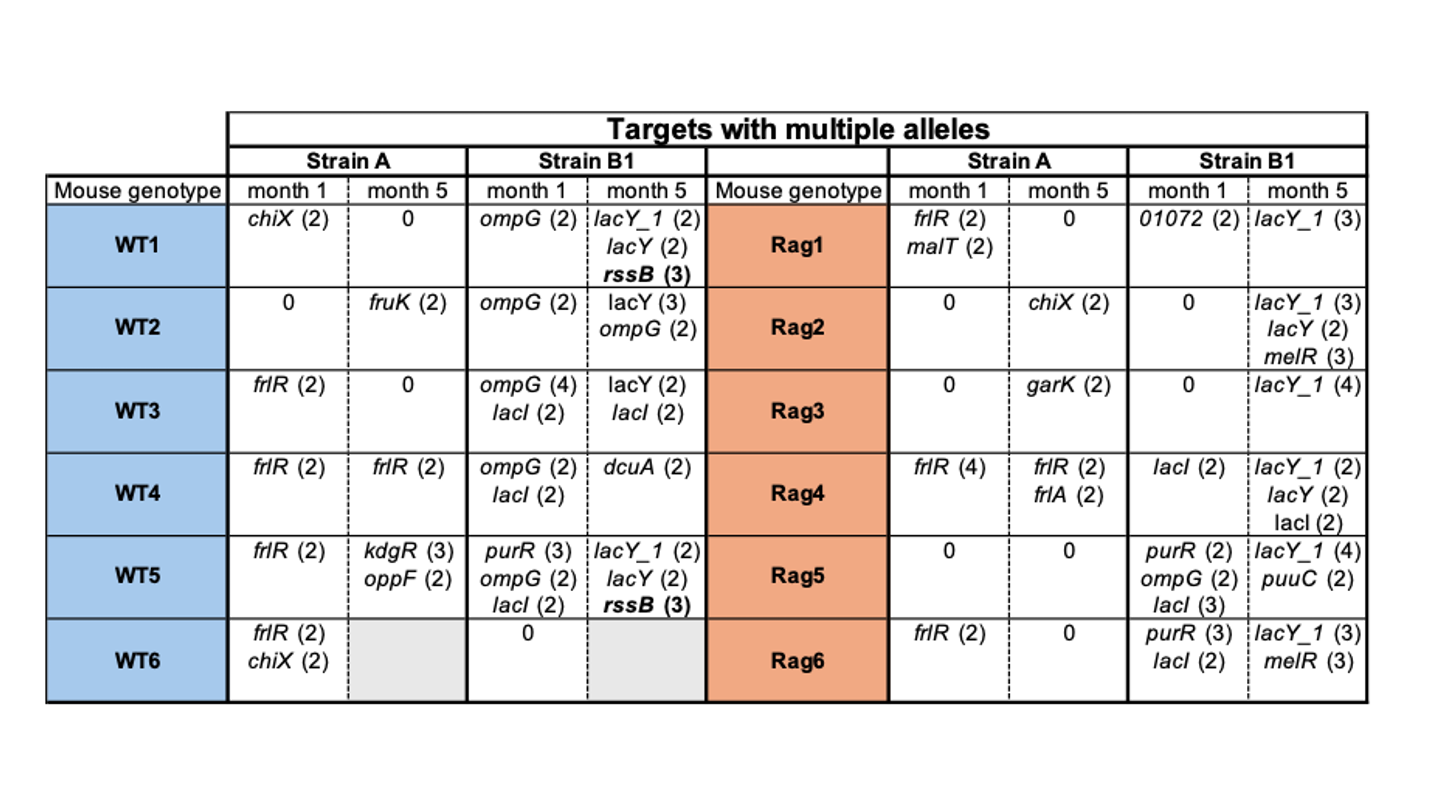

Supplement: S3 Fig — rssB is mutated in strain B1 specifically when colonizing immune-competent mice and shows a high number of multiple alleles segregating in two of mice. (TIF) [file ppat.1012442.s003.tif]

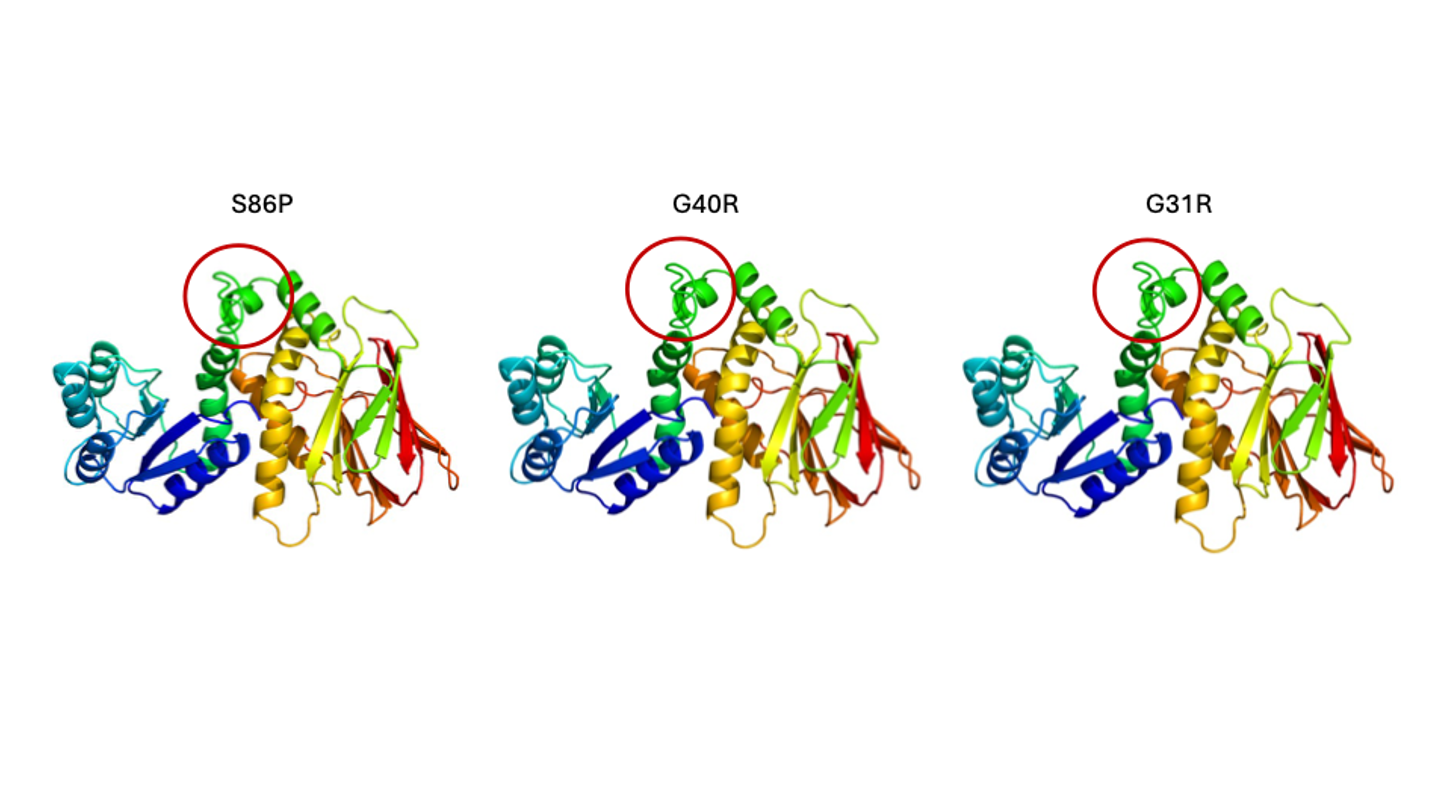

Supplement: S4 Fig — Protein structure prediction was performed using phyre2. (TIF) [file ppat.1012442.s004.tif]

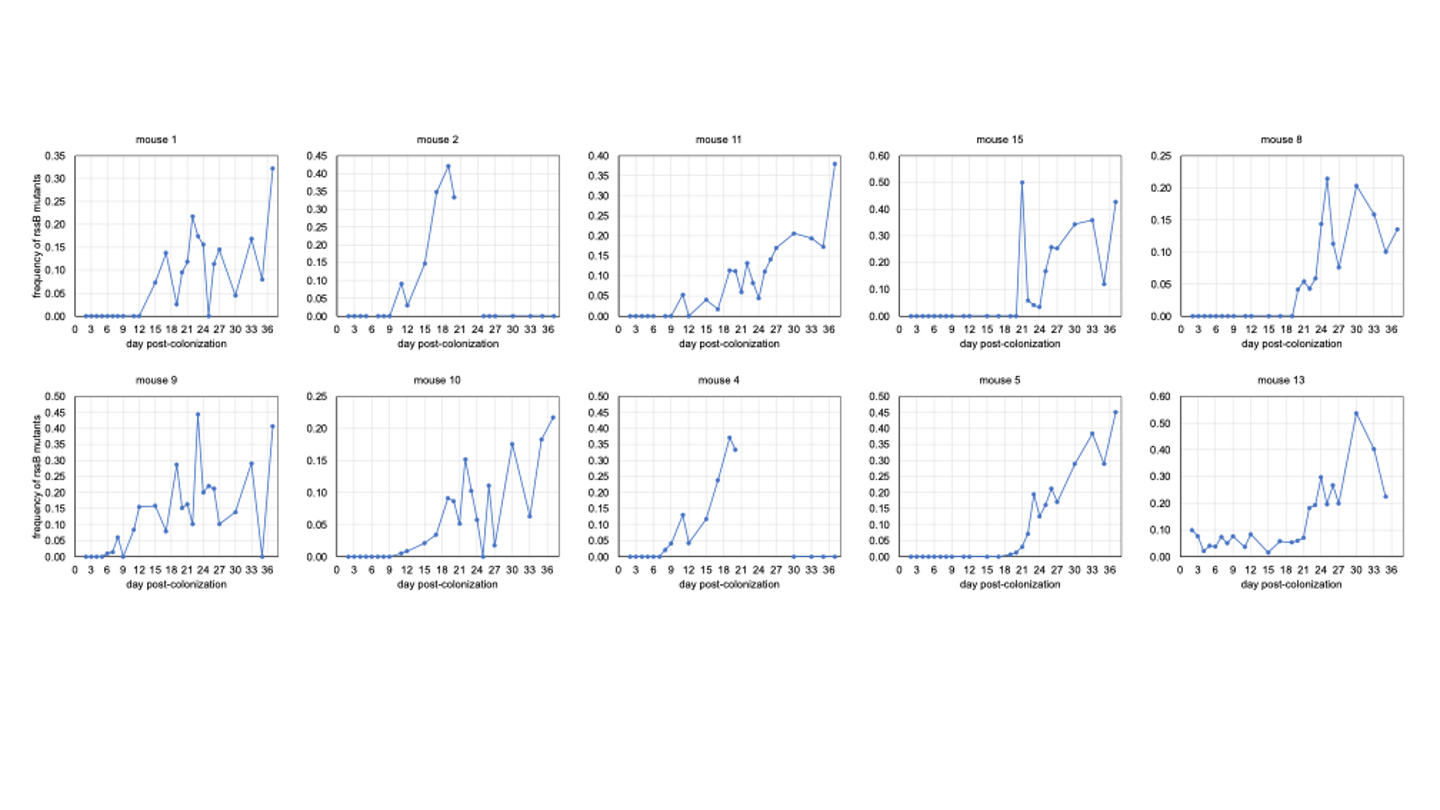

Supplement: S5 Fig — (TIF) [file ppat.1012442.s005.tif]

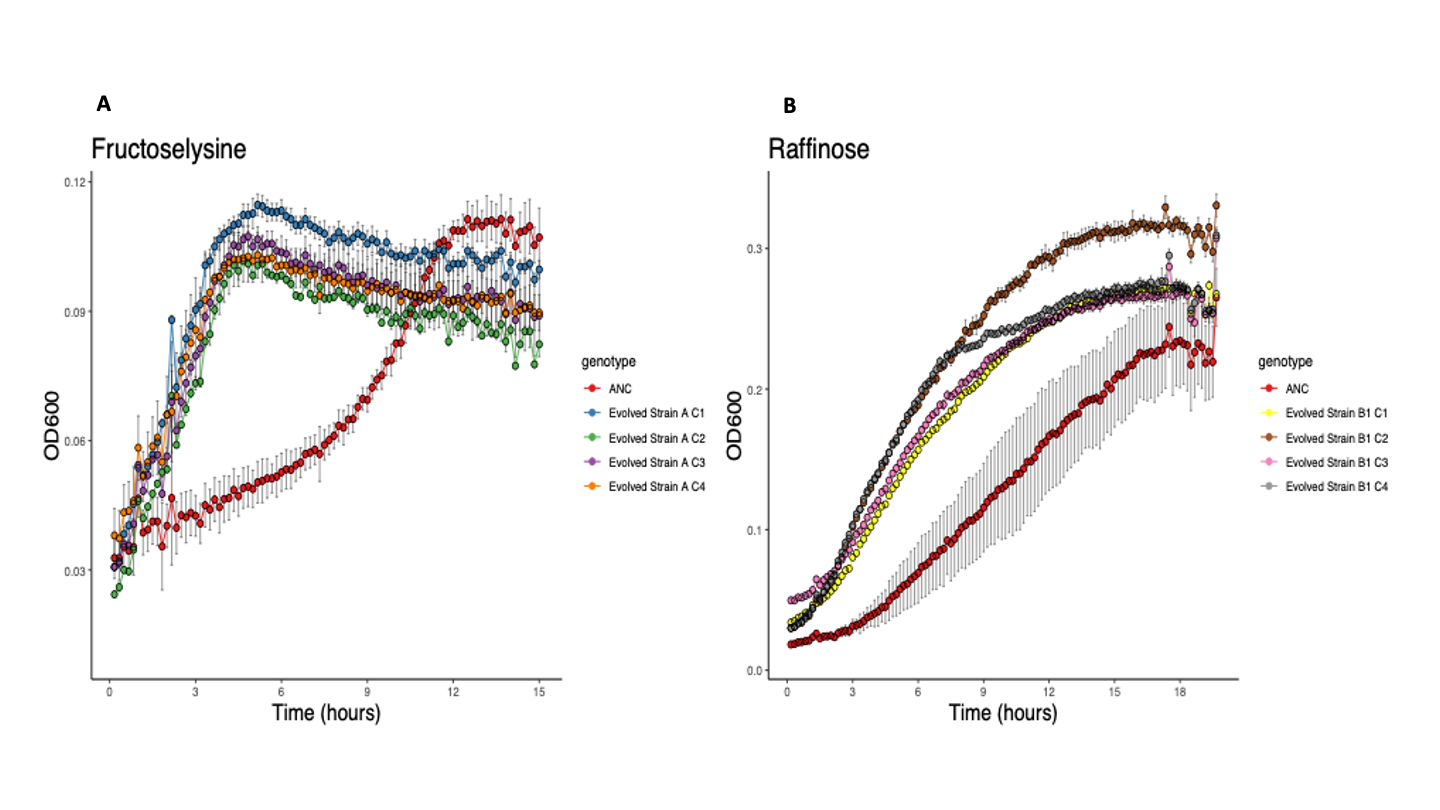

Supplement: S6 Fig — (A) Growth curves in media supplemented with fructoselysine of the ancestral and evolved clones of strain A. (B) Growth curves in media supplemented with raffinose of the ancestral and evolved clones of strain B1. (TIF) [file ppat.1012442.s006.tif]

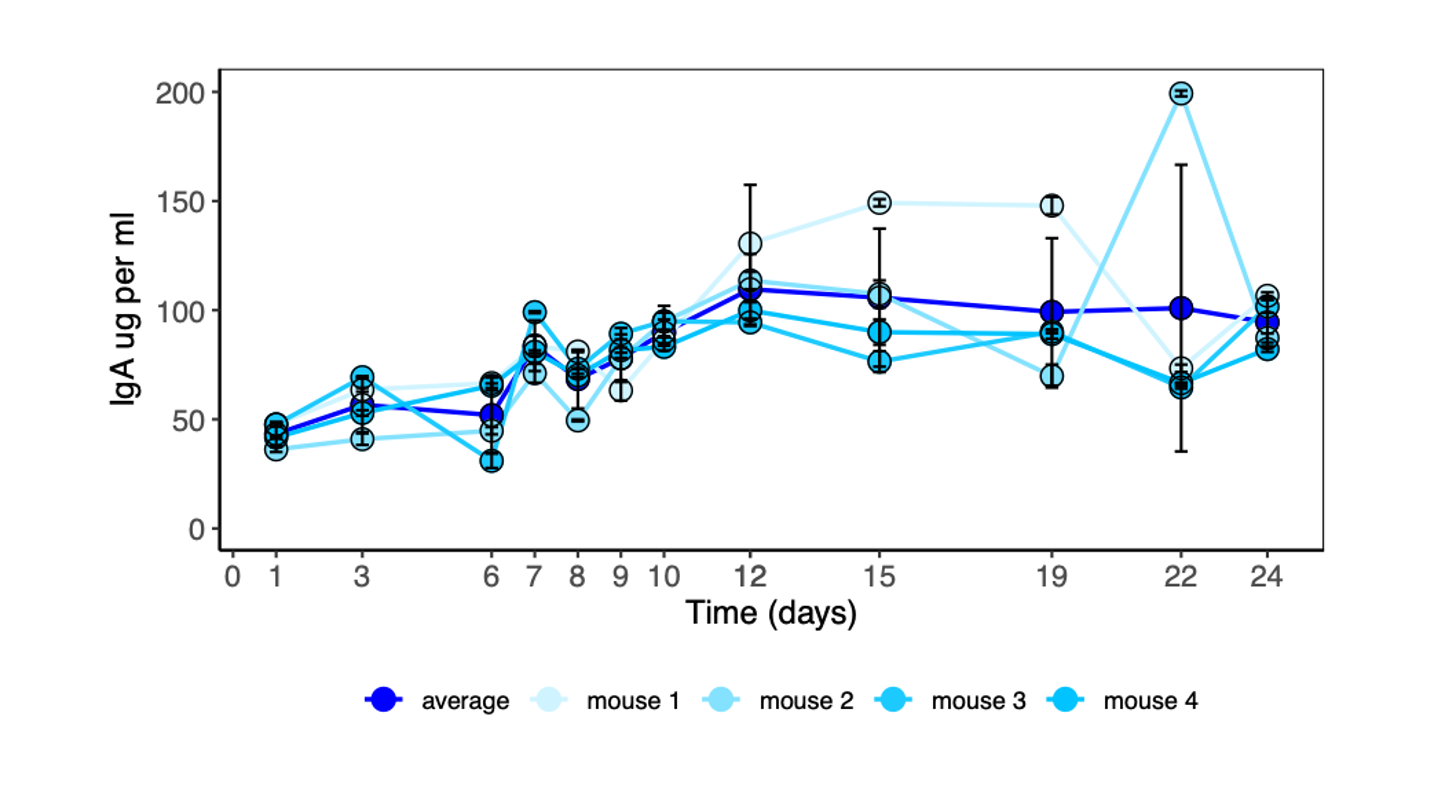

Supplement: S7 Fig — Dark blue circles represent the mean IgA across mice, while light blue circles show the IgA concentration for each individual mice. (TIF) [file ppat.1012442.s007.tif]
