# Supplementary material for: Evolution of Escherichia coli strains under competent or compromised adaptive immunity
Source: PLoS Pathog. 2025 Apr 24;21(4):e1012442. doi: 10.1371/journal.ppat.1012442 (PMC12021133; doi:10.1371/journal.ppat.1012442)
Supplement: S1 Text — (DOCX) [file ppat.1012442.s017.docx]

**Supplementary Text.** We hypothesized that the mutations observed in *rssB* could cause a loss of function of this gene, because one of the mutations observed was a stop codon (Q292*). To test this hypothesis, we first run the sequences (wild-type and variants) on phyre2. It generated a pdb file of each variant protein, visualisation showed modifications on the linker region of the proteins, shown to play an important role on sigma factor S turnover[46] (see Figs 6 and S4). Pdb files were then used for protein structure comparison. A pairwise comparison between each variant and the wild-type predicted structures was performed in Dali protein structure comparison server [52]. When checking protein structure and residues positions, we realized that in all variants from WT5 mice the phenylalanine present in position 140 appeared with a different conformation with the aromatic ring facing the outside of the protein, when compared with the wild-type. Furthermore, when analysing the variants detected in evolved clones of mouse WT1, we observed this phenomenon again (phenylalanine residue to the exterior), with exception of variant L70Q that has a modification (again exposed to the outside of the protein) on the Triptophane residue in position 143. Both modifications at the level of the rssB protein linker region[46]. Phenylalanine is a hydrophobic residue which is nearly always buried inside proteins and plays a role in protein folding due to its bulky, planer and hydrophobic nature, which somewhat reduces flexibility and increases stability of the local conformation [56].
